# Anticancer Activity of a Hexapeptide from Skate (*Raja porosa*) Cartilage Protein Hydrolysate in HeLa Cells

**DOI:** 10.3390/md14080153

**Published:** 2016-08-16

**Authors:** Xin Pan, Yu-Qin Zhao, Fa-Yuan Hu, Chang-Feng Chi, Bin Wang

**Affiliations:** School of Food and Pharmacy, Zhejiang Ocean University, 1st Haidanan Road, Changzhi Island, Lincheng, Zhoushan 316022, China; Uniquepan2015@163.com (X.P.); moonriveryue@163.com (F.-Y.H.); chichangfeng@hotmail.com (C.-F.C.)

**Keywords:** skate (*Raja porosa*), cartilage, peptide, anticancer activity, apoptosis

## Abstract

In this study, the hexapeptide Phe-Ile-Met-Gly-Pro-Tyr (FIMGPY), which has a molecular weight of 726.9 Da, was separated from skate (*Raja porosa*) cartilage protein hydrolysate using ultrafiltration and chromatographic methods, and its anticancer activity was evaluated in HeLa cells. Methylthiazolyldiphenyl-tetrazolium bromide (MTT) assay indicated that FIMGPY exhibited high, dose-dependent anti-proliferation activities in HeLa cells with an IC_50_ of 4.81 mg/mL. Acridine orange/ethidium bromide (AO/EB) fluorescence staining and flow cytometry methods confirmed that FIMGPY could inhibit HeLa cell proliferation by inducing apoptosis. Western blot assay revealed that the Bax/Bcl-2 ratio and relative intensity of caspase-3 in HeLa cells treated with 7-mg/mL FIMGPY were 2.63 and 1.83, respectively, significantly higher than those of the blank control (*p* < 0.01). Thus, FIMGPY could induce apoptosis by upregulating the Bax/Bcl-2 ratio and caspase-3 activation. Using a DNA ladder method further confirmed that the anti-proliferation activity of FIMGPY was attributable to its role in inducing apoptosis. These results suggest that FIMGPY from skate cartilage protein hydrolysate may have applications as functional foods and nutraceuticals for the treatment and prevention of cancer.

## 1. Introduction

Cancer is one of the single most important causes of death in humans, inducing approximately 8.2 million deaths or 14.6% of all human deaths in 2012 [1]. Currently, chemotherapy is the most common method used to eliminate cancer cells, prevent cancer recurrence, control cancer by slowing cell growth, and reduce symptoms [2]. However, healthy cells may be damaged by the many side effects of anticancer drugs, and resistance to anticancer drugs has been observed [3]. Therefore, substantial attention is being paid to identifying anticancer drugs with high efficiency and low toxicity from natural sources [4].

Bioactive peptides, which consist of 2–20 amino acid residues, are inactive in the sequence of their parent proteins and can be released by enzymatic hydrolysis either during gastrointestinal digestion in the body or during food processing. To date, some peptides with anticancer and antioxidant activities have been purified from various protein hydrolysates [5]. (Leu-Ala-Asn-Ala-Lys) LANAK, which has a MW of 515.29 Da and is from oyster protein hydrolysates, was shown to initiate cancer cell death by inhibiting cancer cell growth, increasing DNA damage and apoptosis in the HT-29 colon cancer cell line, and displaying strong antioxidant potential as a 2,2-diphenyl-1-picrylhydrazyl radical (DPPH•) scavenger [1]. (Gln-Pro-Lys) QPK, which was isolated from a sepia ink protein hydrolysate, could significantly inhibit the proliferation of DU-145, PC-3, and LNCaP cells in a time- and dose-dependent manner. This peptide induced apoptosis by decreasing the expression of the anti-apoptotic protein Bcl-2 and increasing the expression of the apoptotic protein Bax [6]. Tyr-Ala-Leu-Arg-Ala-His (YALRAH), which has a MW of 670.77 Da and is from half-fin anchovy (*Setipinna taty*) hydrolysates, exhibited strong anti-proliferation effects on human prostate cancer PC-3 cells, with an IC_50_ of 11.1 μM [7]. Arg-Gln-Ser-His-Phe-Ala-Asn-Ala-Gln-Pro (RQSHFANAQP), which has a MW of 1155 Da and is from chickpea protein hydrolysates, showed significant dose-dependent activities in hydroxyl radical (HO•)-(EC_50_ 2.03 μM), DPPH•-(EC_50_ 3.15 μM) and 2,2′-azino-bis-3-ethylbenzothiazoline-6-sulfonic acid radical (ABTS^+^•)-(EC_50_ 2.31 μM) scavenging assays. Additionally, cell viability assays showed high anti-proliferative activities on the breast cancer cells MCF-7 and MDA-MB-231, with IC_50_ values of 2.38 and 1.50 μmol/mL, respectively. Furthermore, the key tumor suppressor protein (p53) level was shown to increase with increasing RQSHFANAQP concentrations by enzyme-linked immunosorbent assay (ELISA) [8]. WPP, which has a MW of 398.44 Da and is isolated from blood clam (*Tegillarca granosa*), showed significant antioxidant activities against DPPH•, HO•, $O_{2}^{-}$•, and ABTS^+^• with EC_50_ values of 1.388, 0.406, 0.536, and 2.75 mg/mL, respectively. Furthermore, this peptide exhibited strong, dose-dependent cytotoxicity toward PC-3, DU-145, H-1299, and HeLa cell lines and significantly changed the morphologies of PC-3 cells [2]. Previous research indicated that food-derived peptides could have the potential to prevent and treat diseases associated with reactive oxygen species (ROS), specifically cancers [9]. Therefore, consuming antioxidant peptides could dramatically reduce organismal ROS levels and contribute substantially to maintaining health and preventing ROS-associated diseases, especially cancers [10,11,12].

Cartilaginous fishes (Chondrichthyes) are a commercially important species. During processing, large quantities of cartilage are discarded as waste because of their low economic value. In our previous studies, three antioxidant hexapeptides—Phe-Ile-Met-Gly-Pro-Tyr (FIMGPY), Gly-Pro-Ala-Gly-Asp-Tyr (GPAGDY) and Ile-Val-Ala-Gly-Pro-Gln (IVAGPQ)—were isolated from skate (*Raja porosa*) cartilage protein hydrolysates [13], and FIMGPY exhibited good scavenging activities on (DPPH•)-(EC_50_ 3.5768 M), (HO•)-(EC_50_ 4.1821 M), ($O_{2}^{-}$•)-(EC_50_ 3.1181 M) and (ABTS^+^•)-(EC_50_ 1.4307 M), respectively. In addition, FIMGPY showed the higher anti-proliferation activity in HeLa cells than those of GPAGDY and IVAGPQ. Therefore, the objective of the present study was to investigate the anticancer activities and molecular mechanisms of FIMGPY in HeLa cells.

## 2. Results and Discussion

### 2.1. Proliferation Inhibition of HeLa Cell Lines

**Figure 1:**
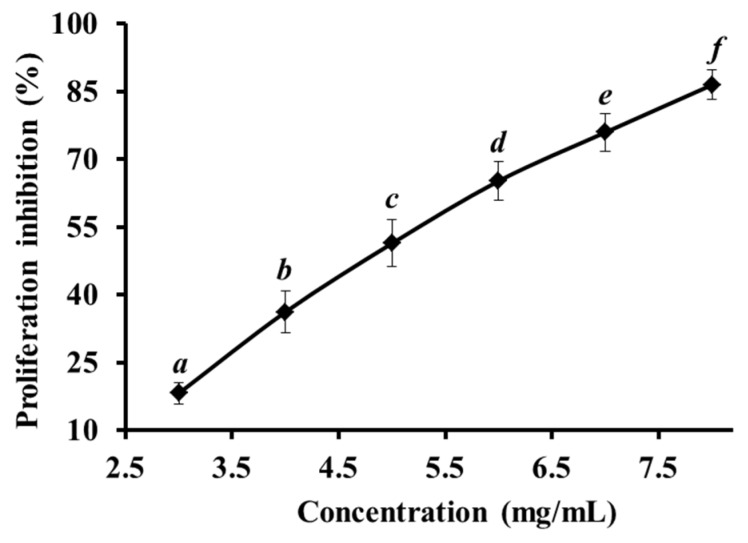
Proliferation inhibition of HeLa cell lines treated by FIMGPY for 24 h. All data are presented as the mean ± standard deviation (SD) of three experiments. ^*a*–*f*^ Values with same letters indicate no significant difference for each group of samples at the same concentration (*p* > 0.05).

Cell proliferation is a physiological process that occurs in almost all tissues and under many circumstances. Under normal conditions, the balance between proliferation and programmed cell death, which usually occurs via apoptosis, is maintained by regulating both processes to ensure the integrity of tissues and organs. However, uncontrolled cell division can induce tissue proliferation and even cancer [14]. Therefore, the inhibition of cell proliferation is thought to be an effective method for tumor therapy. In this study, the HeLa cell line was used to measure the proliferation inhibition rate of FIMGPY. The peptide was evaluated in mouse embryo fibroblast NIH3T3 cells under the same experimental conditions to determine its cytotoxic effect on normal cells. As shown in Figure 1, FIMGPY showed strong, dose-dependent cytotoxicity against HeLa cell lines, with an IC_50_ of 4.81 mg/mL for 24 h. The IC_50_ value of FIMGPY in HeLa cells was lower than those of GPAGDY (4.86 mg/mL) and IVAGPQ (6.26 mg/mL), which are also from skate cartilage protein hydrolysates. The results indicate that FIMGPY exerted higher cytotoxic activity against HeLa cells under identical conditions than the other two peptides. The proliferation-inhibition rate of FIMGPY in NIH3T3 cells (IC_50_ 4.81 mg/mL for 24 h) was also far below than that in HeLa cells (data not shown), suggesting that FIMGPY has almost no cytotoxic effects on normal cells. Therefore, that FIMGPY is cell selective, destroying tumor cells rather than normal cells.

The composition of cell membrane bilayers and the distribution of phospholipids determine cell selectivity and cell susceptibility to lysis. The amount of phosphatidylserine (PS) located in the outer leaflets of cancer cell membranes is 3–7 times that found in the inner leaflets of normal cell membranes [15]. FIMGPY is composed of the hydrophobic amino acids Phe (F), Ile (I), Met (M), and Pro (P), which could lead to increased interactions between FIMGPY and the outer leaflets of tumor cell membrane bilayers, which have high phospholipid contents. These increased interactions may explain FIMGPY’s cell selectivity.

### 2.2. Morphological Observations by Acridine Orange/Ethidium Bromide (AO/EB) Staining

Apoptosis is a process of programmed cell death characterized by biochemical and morphological processes and plays a crucial role in developing and maintaining the health of the body by eliminating old, unnecessary and unhealthy cells [16]. During different stages of apoptosis, some characteristic cell morphologies include blebbing, shrinkage, nuclear fragmentation, chromatin condensation, poly-nucleosomal DNA fragmentation, global mRNA decay, and the fragmentation of cells into apoptotic bodies [17]. Therefore, fluorescence microscopy and AO/EB staining methods were employed to observe the cell changes to distinguish between apoptotic and normal cells, and determine the effects of external factors on cancer cells [6].

**Figure 2:**
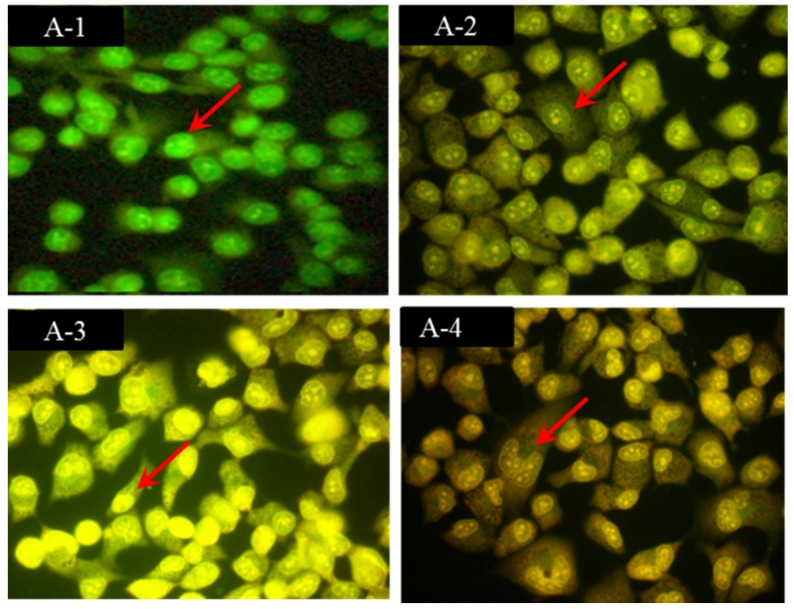
Morphological observation with AO/EB staining at 400× actual magnification. HeLa cells were treated with FIMGPY at (**A-1**) 0, (**A-2**) 3, (**A-3**) 5, and (**A-4**) 7 mg/mL for 24 h. (**A-1**) Cell indicated by the arrow indicates viable cell; (**A-2** and **A-3**) Cells indicated by the arrow indicates early apoptotic cells; (**A-4**) Cell indicated by the arrow indicates late apoptotic cell. Each experiment was performed in triplicate and generated similar morphological features.

**Figure 3:**
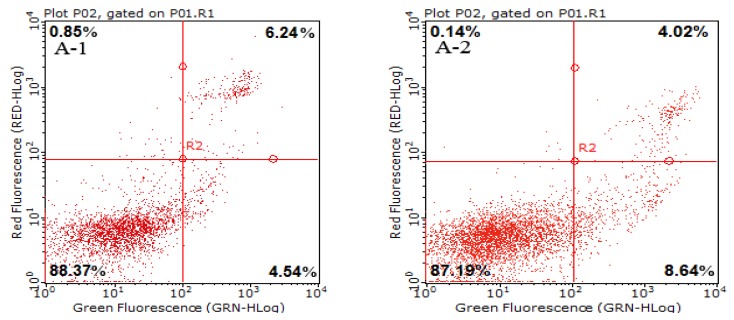

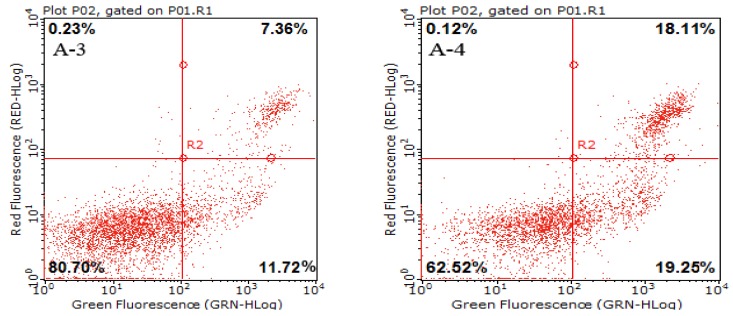
Flow cytometry analysis of HeLa cells by double-labeling with Annexin-V and PI. Quadrants: lower left-live, cells; upper left, necrotic cells; lower right, early apoptotic cells; upper right, late apoptotic cells. The percentages of early apoptotic cells were (**A-1**) 4.54% in the blank control cells; (**A-2**) 8.64% in the 3-mg/mL FIMGPY-treated cells; (**A-3**) 11.72% in the 5-mg/mL FIMGPY-treated cells; and (**A-4**) 19.25% in the 7-mg/mL FIMGPY-treated cells. All data are presented as the mean ± standard deviation (SD) of three experiments.

**Figure 4:**
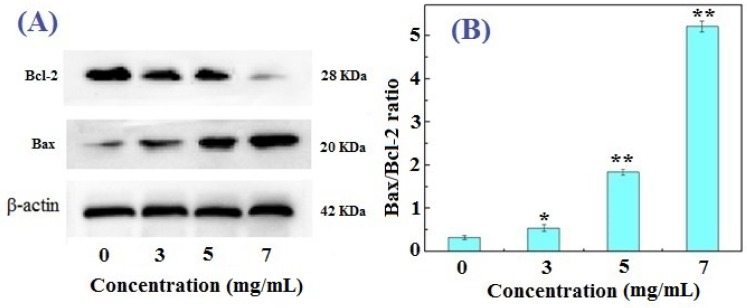
Expression of the apoptosis-associated proteins Bax and Bcl-2 in HeLa cells treated with FIMGPY for 24 h. (**A**) Sodium dodecyl sulfate polyacrylamide gel electrophoresis (SDS-PAGE) patterns for Bax and Bcl-2 and (**B**) the Bax/Bcl-2 ratio. * *p* < 0.05 and ** *p*< 0.01 vs. control.

As shown in Figure 2, HeLa cells showed significant, morphological, apoptotic changes after treatment with 0-, 3-, 5-, and 7-mg/mL FIMGPY for 24 h. Green, yellow/green and reddish/orange staining of the cells indicate viable, early apoptotic, and late apoptotic cells, respectively. The yellow/green staining in Figure 2(A-2,A-3) shows HeLa cells that was at an early stage of apoptosis. Typical apoptotic changes, such as condensed chromatin, cytoplasmic blebs, and fragmented nuclei, were also observed in the HeLa cells after exposure to 3- and 5-mg/mL FIMGPY for 24 h. In Figure 2(A-4), additional features—i.e., orange necrotic cell apoptotic bodies—were observed, indicating that the HeLa cells were in the final stages of apoptosis after exposure to 7-mg/mL FIMGPY for 24 h. The AO/EB staining results revealed that the morphological features of the apoptotic HeLa cells were dose dependent, similar to previous AO/EB staining results obtained for DU-145 and PC-3 cells treated with QPK from cuttlefish ink [6], PC-3 cells treated with Arg-Ala-Ala-Leu-Ala-Val-Val-Leu-Gly-Arg-Gly-Gly-Pro-Pro (RAALAVVLGRGGPR) and Arg-Asp-Gly-Asp-Ser-Cys-Arg-Gly-Gly-Gly-Pro-Val (RDGDSCRGGGPV) from *Bullacta exarata* [18], and PC-3 cells treated with Trp-Pro-Pro (WPP) from blood clam [2].

### 2.3. Cell Apoptotic Rate Detected by Flow Cytometry

In normal cells, PS distributes only on the inner side of the cytomembrane and transfers to the outer side of the cytomembrane during early cell apoptosis. Therefore, Annexin V can bind to PS that is expressed on the outer layer of the cytomembrane and is used to identify cells entering apoptosis [19]. Propidium iodide (PI) is used as a DNA stain for flow cytometry to evaluate cell viability or DNA content via cell cycle analysis and to differentiate necrotic, apoptotic, and normal cells [20]. Thus, Annexin V-fluorescein isothiocyanate (FITC)/PI can identify distinct cell stages and quantitatively illustrate the apoptotic process [21].

The percentages of Annexin V-stained HeLa cells treated with FIMGPY at concentrations ranging from 3 to 7 mg/mL are depicted in Figure 3. The percentage of Annexin V-stained HeLa cells was 4.54% for the control. After 24 h of exposure to FIMGPY, the apoptosis percentages increased to 8.64 ± 0.31, 11.72 ± 0.57 and 19.25 ± 0.76% for concentrations of 3, 5, and 7 mg/mL, respectively. Compared with the control, the apoptotic effect on the HeLa cells markedly increased as the FIMGPY concentration increased. Therefore, FIMGPY displayed a high capacity to induce apoptosis in HeLa cells.

### 2.4. Western Blotting Results for Bcl-2, Bax, and Caspase-3 in FIMGPY-Treated HeLa Cells

The flow cytometry assay indicated that the apoptosis rate increased in HeLa cells as the FIMGPY concentration increased. Apoptosis is a highly regulated and controlled process that confers advantages during an organism’s life cycle. Therefore, the initiation of apoptosis is precisely regulated by activation mechanisms involving specific factors; for example, caspases and Fas receptors promote apoptosis, whereas some members of the Bcl-2 family of proteins inhibit apoptosis [22]. To further confirm the effects of FIMGPY in HeLa cells and explain the reasons for the observed apoptosis, western blot assay was performed to investigate anti- and pro-apoptosis protein expression levels in treated HeLa cells.

Two distinct pathways (intrinsic and extrinsic) can lead to the activation of apoptosis. The intrinsic or mitochondrial apoptosis is crucially regulated by the interplay/balance between the pro- and anti-apoptotic Bcl-2 family members. Consequently, the Bcl-2 family proteins play a pivotal role in determining whether a cell will live or die [23,24]. Members of the Bcl-2 family, such as Bax, Bak, Bad, and Bcl-Xs, possess pro-apoptotic characteristics, whereas other members, such as Bcl-2, Bcl-XL, Bcl-W, Bfl-1, and Mcl-1, act as anti-apoptotic regulators. The apoptosis-inducing effect is more dependent on the balance between Bcl-2 and Bax than on Bcl-2 alone. Typically, the ratio of Bcl-2 and Bax protein expression is used as an index for apoptosis [6]. In this experiment, the levels of pro-apoptotic Bax and anti-apoptotic Bcl-2 proteins were measured by Western blot analysis in the presence of different doses of FIMGPY (0, 3, 5, and 7 mg/mL). As shown in Figure 4, a remarkable upregulation of Bax protein levels and a decrease in the Bcl-2 protein levels were observed as the FIMGPY concentration increased (Figure 4A), eventually leading to an increase in the Bax/Bcl-2 ratio in FIMGPY-treated HeLa cells (Figure 4B). The Bax/Bcl-2 ratio in HeLa cells treated with 7-mg/mL FIMGPY was 2.63, which was significantly higher than that of the blank control (*p* < 0.01). The result indicated that FIMGPY could promote apoptosis in HeLa cells by upregulating the Bax/Bcl-2 ratio.

**Figure 5:**
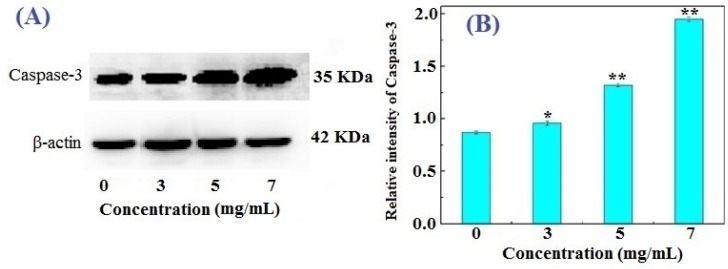
Caspase-3 expression in HeLa cells treated with FIMGPY for 24 h. (**A**) SDS-PAGE pattern of caspase-3 and (**B**) the relative intensity of caspase-3. * *p* < 0.05 and ** *p* < 0.01 vs. control.

Caspases are the executioners of apoptosis and are divided into the following two types according to their functions in apoptosis: (1) initiator (apical) caspases and (2) effector (executioner) caspases [25,26]. Initiator caspases (e.g., caspase-2, 8, 9, and 10) cleave inactive pro-forms of effector caspases, thereby activating them; effector caspases (e.g., caspase-3, 6, 7), in turn, cleave other protein substrates within the cell to trigger apoptosis. Among them, caspase-3 interacts with caspase-8 and caspase-9 in apoptosis, and plays a central role in the execution phase of apoptosis [22,27]. Figure 5A shows that FIMGPY noticeably upregulated caspase-3 levels in HeLa cells and that its relative intensity increased from 0.72 to 1.83 when the peptide concentration ranged from 0 to 7 mg/mL. The relative intensity of caspase-3 at 8 mg/mL was significantly higher than that of the blank control (*p* < 0.01) (Figure 5B). Slee, Adrain, and Martin reported that caspase-3 is the primary executioner caspase in apoptotic death and is necessary for the cytochrome c/dATP-inducible cleavage of fodrin, gelsolin, and U1 small nuclear ribonucleoprotein and DNA fragmentation factor 45/inhibitor of caspase-activated DNase [28]. Caspase-3 is also essential for apoptosis-associated chromatin margination, DNA fragmentation, and nuclear collapse in this system. Therefore, based on the activation of caspase-3, the FIMGPY-induced apoptosis of HeLa cells seemed to be related to the mitochondria-mediated pathway. Therefore, the apoptotic signal will be amplified step by step and the apoptotic process promoted as the caspase-3 level increases.

### 2.5. DNA Ladder Analysis

**Figure 6:**
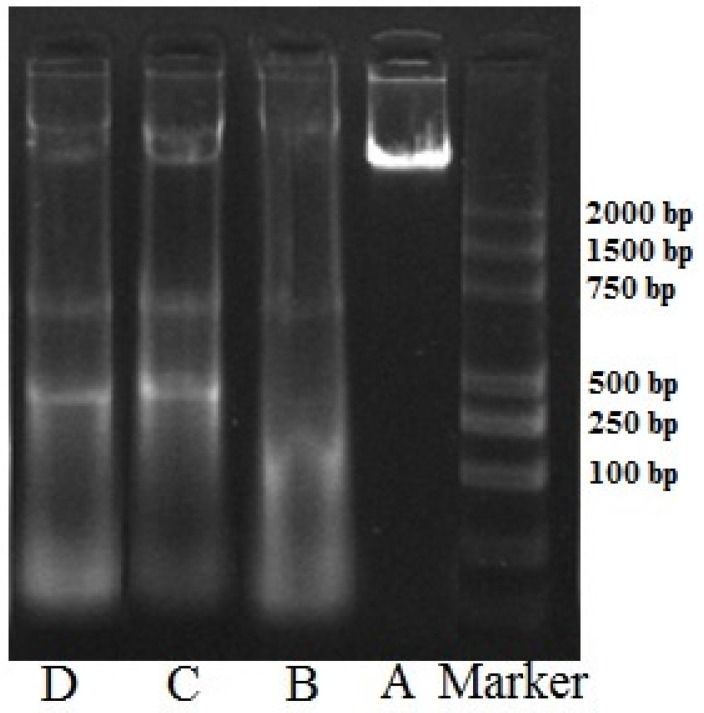
DNA fragmentation assay of HeLa cells treated with different concentrations of FIMGPY for 24 h. (**A**) Blank control; (**B**) 3 mg/mL; (**C**) 5 mg/mL; (**D**) 7 mg/mL, and the MV 2000 marker.

The degradation of nuclear DNA into nucleosomal units is one of the hallmarks of apoptotic cell death. During this process, chromatin DNA is cleaved into inter-nucleosomal fragments, which will show a ladder pattern in agarose gel electrophoresis; thus, apoptosis can be detected via a DNA laddering assay [29,30]. As shown in Figure 6, the DNA bands from the control group of HeLa cells remained intact, whereas DNA ladder patterns were observed for the HeLa cells treated with different concentrations of FIMGPY for 24 h. DNA fragmentation also increased as the FIMGPY concentration increased. These results indicated that FIMGPY could induce apoptosis in HeLa cells and that the number of apoptotic cells increases as the FIMGPY concentration increases. This finding is in good agreement with the AO/EB staining, flow cytometry, and Western blotting analysis results.

### 2.6. Discussion

The structural properties can provide effective guides for evaluating food proteins as potential precursors of bioactive peptides, and design the rational enzymolysis technology to prepare the bioactive peptides from various food-resources proteins [13]. At present, there is still a shortage of solid evidence to clarify the relationship between structural properties of peptides and their anticancer property. However, hydrophobicity, molecular size, amino acid composition, and sequence are deemed to play an essential role in bioactivity of peptides [31,32]. Molecular size ranged from 0.5 to 3 kDa has been supposed to be a key factor affecting the bioactivity of oxidant activity of protein hydrolysates and peptides [33]. CPe-III (RQSHFANAQP) with a MW of 1155 Da showed high inhibition activity on MCF-7 and MDA-MB-231 cells with EC_50_ of 2.38 and 1.50 μM. QPK with a MW of W 387.4 Da could significantly inhibit the proliferation of DU-145, PC-3, and LNCaP cells in a time- and dose-dependent manner [6]. Therefore, the anticancer activity of FIMGPY might be due to its small molecules (MW 726.9 Da).

In addition, hydrophobic properties could play an important role in their anticancer activities. For example, hydrophobic peptide fractions separated from anchovy sauce have been shown to exhibit cancer-chemopreventive effects in human lymphoma cells (U937) by inducing apoptosis in cancer cells; Ala (A) and Phe (F) were supposed to be the key factors underlying this activity [34,35]. Hydrophobic Ala (A) and Leu (L) residues in the peptide YALPAH were confirmed to be important for this peptide’s anti-proliferative activities in PC-3 cells [7]. Chi et al. reported that the hydrophobic residues Trp (W) and Pro (P) in WPP play a vital role in its proliferation-inhibition ability in PC-3 cell lines. Therefore, Phe (F), Ile (I), Met (M), Pro (P), and Tyr (Y) in the sequence of FIMGPY should contribute to its high anticancer activities [2].

## 3. Experimental Section

### 3.1. Chemicals and Reagents

Skates (*R. porosa*) were purchased from Nanzhen market in Zhoushan City, China. NIH3T3 and HeLa cell lines were purchased from the China Cell Bank of the Institute of Biochemistry and Cell Biology in Shanghai, China. Methylthiazolyldiphenyl-tetrazolium bromide (MTT) and Annexin V-FITC Apoptosis Detection Kits were purchased from Sigma-Aldrich Trading Co., Ltd. (Shanghai, China). All other chemicals and reagents were of analytical grade and were obtained from Sinopharm Chemical Reagent Co., Ltd. (Shanghai, China).

### 3.2. Preparation of Hexapeptide FIMGPY

The hexapeptide FIMGPY, which has a molecular weight of 726.9 Da, was separated from skate (*Raja porosa*) cartilage protein hydrolysate according to the method of Pan et al. [13].

### 3.3. Anti-Tumor Activity

#### 3.3.1. Anti-Proliferative Activity

Anti-proliferative activity was evaluated in vitro by MTT assay using the method of Chi et al. and expressed as IC_50_ values (defined as the concentration of peptide that caused 50% cell death) [2]. Briefly, cells were seeded at a density of 1 × 10^4^ cells per well in a 96-well plate for 24 h at 37 °C in a 5% CO_2_ incubator. Then, the cells were treated with FIMGPY at final concentrations of 3, 4, 5, 6, 7, and 8 mg/mL. Untreated cells were used as a negative control. The cell proliferation-inhibition rate (%) was calculated as follows:

Inhibition rate (%) = [(A_control_ − A_treated_)/(A_control_ − A_blank_)] × 100%

#### 3.3.2. Morphological Study with Fluorescence Microscopy

Apoptosis morphology was evaluated using AO/EB fluorescence staining [6]. Briefly, HeLa cells were seeded in a six-well plate (1 × 10^5^ cells/well) and incubated overnight before treatment. Then, the cells were exposed to FIMGPY at concentrations of 3, 5, and 7 mg/mL for 24 h. Untreated cells served as the negative control. After the designated time, 25 μL of 100-µg/mL AO/EB dye mixture in PBS (pH 7.4) was added to the FIMGPY-treated cells. After staining, the cells were immediately visualized and imaged under a fluorescence microscope (Leica DM 3000, Leica Microsystems, Wetzlar, Germany). Each image was collected with excitation at 488 nm and emission at 520 nm.

#### 3.3.3. Flow Cytometry Analysis

The apoptosis rate was quantitatively detected with an Annexin V-FITC/PI double-staining assay using a FACS Calibur flow cytometer (Becton Dickinson, New York, NY, USA) [2]. Annexin-V binding was performed using an Annexin-V-FITC kit as described by Sigma-Aldrich Trading Co., Ltd. (Shanghai, China). Briefly, HeLa cells were seeded at a density of 1 × 10^5^ cells/well in six-well plates for 24 h and then treated with FIMGPY at concentrations of 3, 5, and 7 mg/mL for 24 h. Then, 1 × 10^5^ cells were collected by centrifugation at 9000× *g* for 5 min at 4 °C, rinsed twice with cold PBS (pH 7.0), gently re-suspended in 400 μL of binding buffer, and incubated with 5 μL of Annexin V-FITC for 15 min and 5 μL of PI (100 μg/mL) for 5 min in the dark. Finally, the cells were analyzed with a flow cytometer. Data analysis was performed with BD FACStation Software (Becton Dickinson, New York, NY, USA). Apoptosis was quantitatively confirmed by analyzing the percentage of early apoptotic cells using Annexin-V-FITC/PI double staining.

#### 3.3.4. Western Blot Analysis

Western blot analysis was conducted according to the method of Huang et al. [6]. HeLa cells were seeded at a density of 1 × 10^5^ cells/well in six-well plates for 24 h and then treated with FIMGPY at concentrations of 3, 5, and 7 mg/mL for 24 h; cell culture medium was used as the negative control. Then, 1 × 10^5^ cells were collected by centrifugation at 9000× *g* for 5 min at 4 °C and rinsed twice with cold PBS (pH 7.2). The cells from the six-well plates were treated with 200 μL of lysis buffer containing phenylmethanesulfonyl fluoride for 30 min. Subsequently, the treated cells were centrifuged for 5 min at 12,000× *g*, and the protein in the supernatant was measured by bicinchoninic acid (BCA) assay and separated by SDS-PAGE. After SDS-PAGE, proteins were transferred to a polyvinylidene difluoride (PVDF) membrane, and the membrane was blocked with 10% non-immune serum for 2 h and then incubated with primary antibody (Cell Signaling, rabbit monoclonal antibody, 1:1000) overnight at 4 °C. After washing three times with Tris-buffered saline with 0.1% Tween-20 (TBST) buffer, the membrane was incubated with the secondary antibody (goat-anti-rabbit horseradish peroxidase [HRP]-conjugated 1:3000) at room temperature for 2 h and subsequently washed with TBST. The intensity of the specific immunoreactive bands was detected by enhanced chemiluminescence (ECL), quantified by densitometry and expressed as a ratio to β-actin.

#### 3.3.5. DNA Ladder Analysis

HeLa cells were seeded at a density of 1 × 10^5^ cells/well in six-well plates for 24 h and then treated with FIMGPY at concentrations of 3, 5, and 7 mg/mL for 24 h; cell culture medium was used as the negative control. The cells were collected by centrifugation at 9000× *g* for 5 min at 4 °C, rinsed twice with cold PBS (pH 7.2), and treated with 500 μL of lysis buffer at 50 °C. After 12 h, an equal volume of phenol-chloroform-isoamyl alcohol was added, and the solution was mixed gently and centrifuged at 12,000× *g* for 10 min. Then, the aqueous phase was transferred to new Eppendorf tubes. An equal volume of cold chloroform-isoamyl alcohol was added to the tubes and mixed gently by inversion. The aqueous phase was once again transferred to new Eppendorf tubes, treated with 60 μL of ammonium acetate (10 M) and 600 μL of absolute ethanol, and then stored at −20 °C. After 12 h, the solution was centrifuged at 12,000× *g* for 10 min, and the precipitate was collected and air-dried for 30 min. Then, the dried DNA was dissolved in 40 μL of Tris-ethylenediaminetetraacetic acid (EDTA) (TE) buffer (pH 7.4) and electrophoresed on a 1.0% agarose gel. The gel was examined and photographed with an ultraviolet gel documentation system (iNTAS, Goettingen, Germany).

### 3.4. Statistical Analysis

The results are presented as the mean ± SD (*n* = 3). An ANOVA test using SPSS 19.0 (Statistical Program for Social Sciences, SPSS Corporation, Chicago, IL, USA) was used to analyze the experimental data. Significant differences were determined using Duncan’s multiple-range test (*p* < 0.05 and 0.01).

## 4. Conclusions

In this study, the anticancer activities of the skate (*R. porosa*) cartilage protein hydrolysate peptide FIMGPY were evaluated in HeLa cells. FIMGPY displayed high anti-proliferation activities in HeLa cells, inducing apoptosis by upregulating the Bax/Bcl-2 ratio and caspase-3 activation. Thus, FIMGPY has great potential as an anti-carcinogen in the food and pharmaceutical industries. However, studies on the structure-activity relationship of bioactive peptides and in vivo studies on this peptide’s anticancer activities remain to be performed.
